# Supplementary material for: DHX9 SUMOylation is required for the suppression of R-loop-associated genome instability
Source: Nat Commun. 2024 Jul 17;15:6009. doi: 10.1038/s41467-024-50428-4 (PMC11255299; doi:10.1038/s41467-024-50428-4)
Supplement: Supplementary file 1 — Supplementary Information [file 41467_2024_50428_MOESM1_ESM.pdf]

Supplementary information

DHX9 SUMOylation is required for the suppression of R-loop-associated genome instability

Bing-Ze Yang, Mei-Yin Liu, Kuan-Lin Chiu, Yuh-Ling Chien, Ching-An Cheng, Yu-Lin Chen, Li-Yu Tsui, Keng-Ru Lin, Hsueh-Ping Catherine Chu, and Ching-Shyi Peter Wu

Correspondence to: cswu2017@ntu.edu.tw

This file includes:

Supplementary Figures 1-8

Supplementary Tables 1-5

Supplementary methods

a

SUMOylation site prediciton by SUMOplot

Position	Group	Score
K76	VRINEIKSEEVPAF	0.94
K365	QISMDLKNELMYQL	0.91
K120	PPHLALKAENNSEV	0.91

b

SUMOylation site prediciton by GPS-SUMO

Position	Peptide	Score
5	***MGDVKNFL	0.89
76	LVRINEIKSEE	0.86
120	LPPHLALKAEN	0.86

c

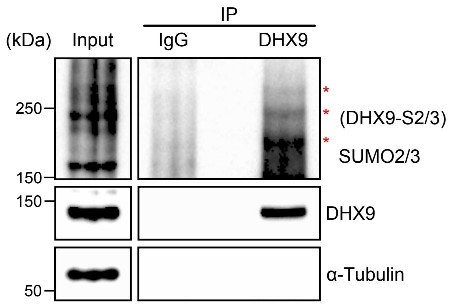

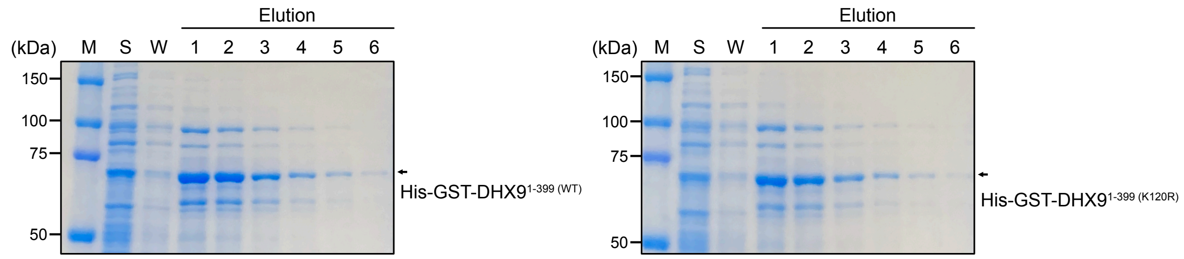

Supplementary Fig. 1 K120 is a major SUMO conjugation site of DHX9.

a, b Potential SUMOylation sites on DHX9 predicted by two distinct web-based computational programs. SUMOplot analysis program, https://www.abcepta.com/sumoplot (**a**) and GPS-SUMO 6.0 (**b**). Only the top 3 candidate sites were selected shown. **c** SUMOylation of endogenous DHX9 by SUMO2/3 was determined in HeLa cells. Representative data of three biological experiments shows SUMO2/3 conjugation of DHX9. Asterisks indicate SUMO2/3 conjugated DHX9. **d, e** Expression and purification of recombinant $DHX9^{WT}$ and $DHX9^{K120R}$ fragments were determined by SDS-PAGE and Coomassie blue staining. Purified His-GST-DHX9 fragments were used to test DHX9 SUMOylation *in vitro*. The black arrow indicates the predicted molecular weight of recombinant His-GST-DHX9 (M, protein marker; S, supernatant of bacterial lysate; W, wash buffer). Source data are provided as a Source Data file.

a

UniProt ID	Sequence		Species
Q08211	DTTANAEGDLPTTMGGPLPPHLALKAE---N-NSE----VGASGY-GVPGPTWDRGANLKDY	148	***Homo sapiens*** (HUMAN)
H2Q0R0	DTTANAEGDLPTTMGGPLPPHLALKAE---N-NSE----VGASGY-GVPGPTWDRGANLKDY	148	***Pan troglodytes*** (CHIMPANZEE)
O70133	DSTASAAEGLPAPMGGPLPPHLALKAEENN--SGV----ES-SGY-GSPGPTWDRGANLKDY	150	***Mus musculus*** (MOUSE)
D4A9D6	DSTASTGEGLPPPMGGPLPPHLALKAAENN--SGV----ESSSGY-GSPGPTWDRGANLKDY	151	***Rattus norvegicus*** (RAT)
F6UTW6	DTA-NTGGGLPTTMGGPLPPHLALKAE---N-NSG----VGASGY-GVPGPTWDRGANLKDY	148	***Equus caballus*** (HORSE)
A0A8I3RTG4	DSAATMGGGLPTTMGGPLPPHLALKAE---N-NSG----IGASGY-GPPGPTWDRGANLKDY	149	***Canis lupus familiaris*** (DOG)
G3UKE5	DGTASTDGSLPTTMGGPLPPHLALKAEGEKN-NSG----VGASGY-GVPGPTWDRGANLKDY	152	***Loxodonta africana*** (AFRICAN ELEPHANT)
K7GND3	DDTANAGGGLPATMGGPLPPHLALKAENN-------------SGY-GVTGPTWDRGANLKDY	144	***Sus scrofa*** (PIG)
A0A452F5Z4	DTT-TDDGGLPGNLGGPLPPHLTLQAE---N-NSG----GGGSGY----VPTWDRGANLKDY	145	***Capra hircus*** (GOAT)
H9G9V0	EA-GGAAEEGNFPLGGPIPPHLAIKTETDGAQPTG----IGISGY---GGAQWDRGANLKDY	150	***Anolis carolinensis*** (GREEN ANOLE)
Q68FK8	DGASLKSEGFSGPTGGPLPPHLSLQAESSGAAPMRSNTGFNPPAY-G-GGAQWERGANLKDY	151	***Xenopus laevis*** (AFRICAN CLAWED FROG)
A0A8M1P3F9	DDG---GGFGNLPSNCPLPPHLAVKKELESCEGSAPVPGVTGLGYSGRNNAPWERGANLQEY	155	***Danio rerio*** (ZEBRAFISH)

b

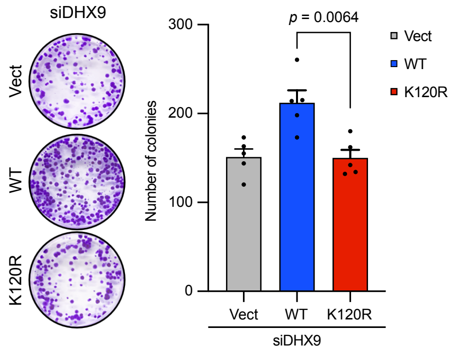

c

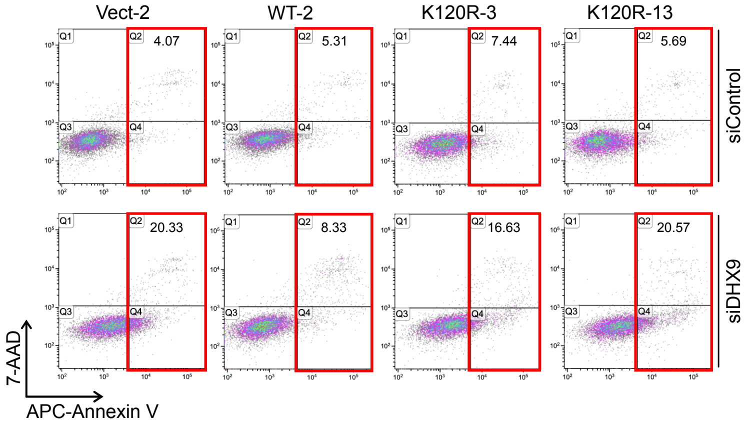

Supplementary Fig. 2 DHX9 SUMOylation is crucial for cell survival.

a DHX9 protein sequence alignment across different vertebrates. The consensus SUMOylation motif is underlined, and the conserved K120 across different species is highlighted in red. **b** Effects of transiently expressed SFB-DHX9^{WT} and SFB-DHX9^{K120R} on colony formation in HeLa cells transfected with DHX9 siRNA (mean ± SEM with dots indicating results of n=5, two-sided *t*-test). **c** Representative images of Annexin V-APC apoptosis analysis from three independent experiments, as shown in Fig. 2e. Annexin V-APC-positive cells within the red boxes (Q2/Q4 quadrants) were quantified. Source data are provided as a Source Data file.

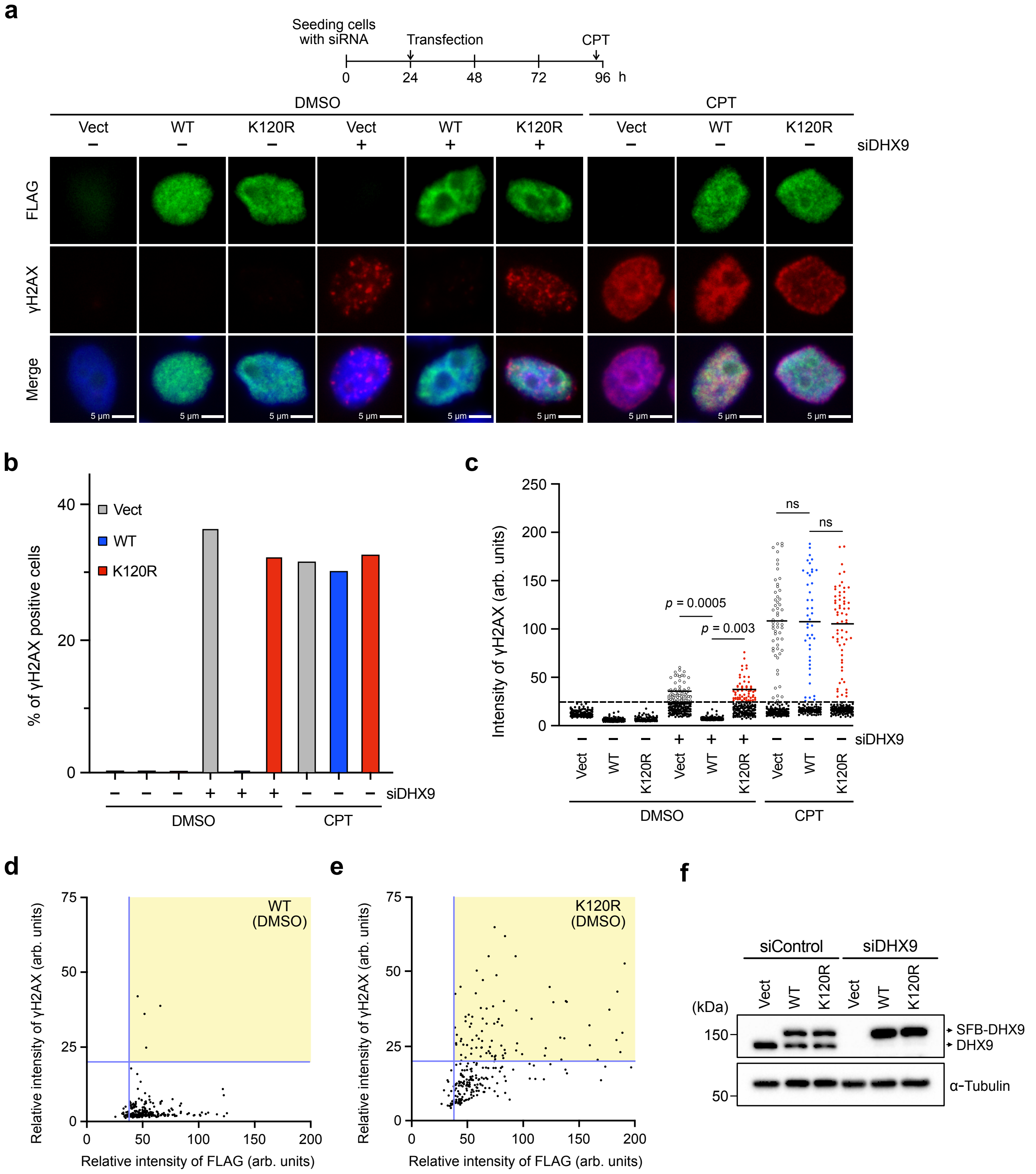

Supplementary Fig. 3 DHX9 SUMOylation prevents the accumulation of DNA damage.

a-e HeLa cells with DHX9 depleted by siRNA were transiently transfected with the corresponding empty vector, siDHX9-resistant SFB-DHX9^{WT}, or SFB-DHX9^{K120R} for 3 d, followed by treatment with DMSO or CPT (1 μM) for 1 h. The percentage and intensity of γH2AX (red) and FLAG (green) staining were determined. **a** Representative images of two independent experiments. Scale bar = 5 μm. **b** Representative data of the percentage of γH2AX foci-positive cells of n = 2 independent experiments. **c** Intensity of γH2AX foci was quantified in DMSO or CPT-treated HeLa cells. Representative data of n = 2 biological replicates analyzed by two-sided *t*-test. Cells with background staining were colored black. The black line indicates the median. **d, e** The correlation between SFB-DHX9 variant expression and γH2AX foci intensity in cells expressing WT or K120R DHX9 treated with DMSO were plotted in 2D, respectively. **f** Representative Western blot of two biological experiments shows the levels of endogenous and SFB-tagged DHX9 in different HeLa derivative lines. Source data are provided as a Source Data file.

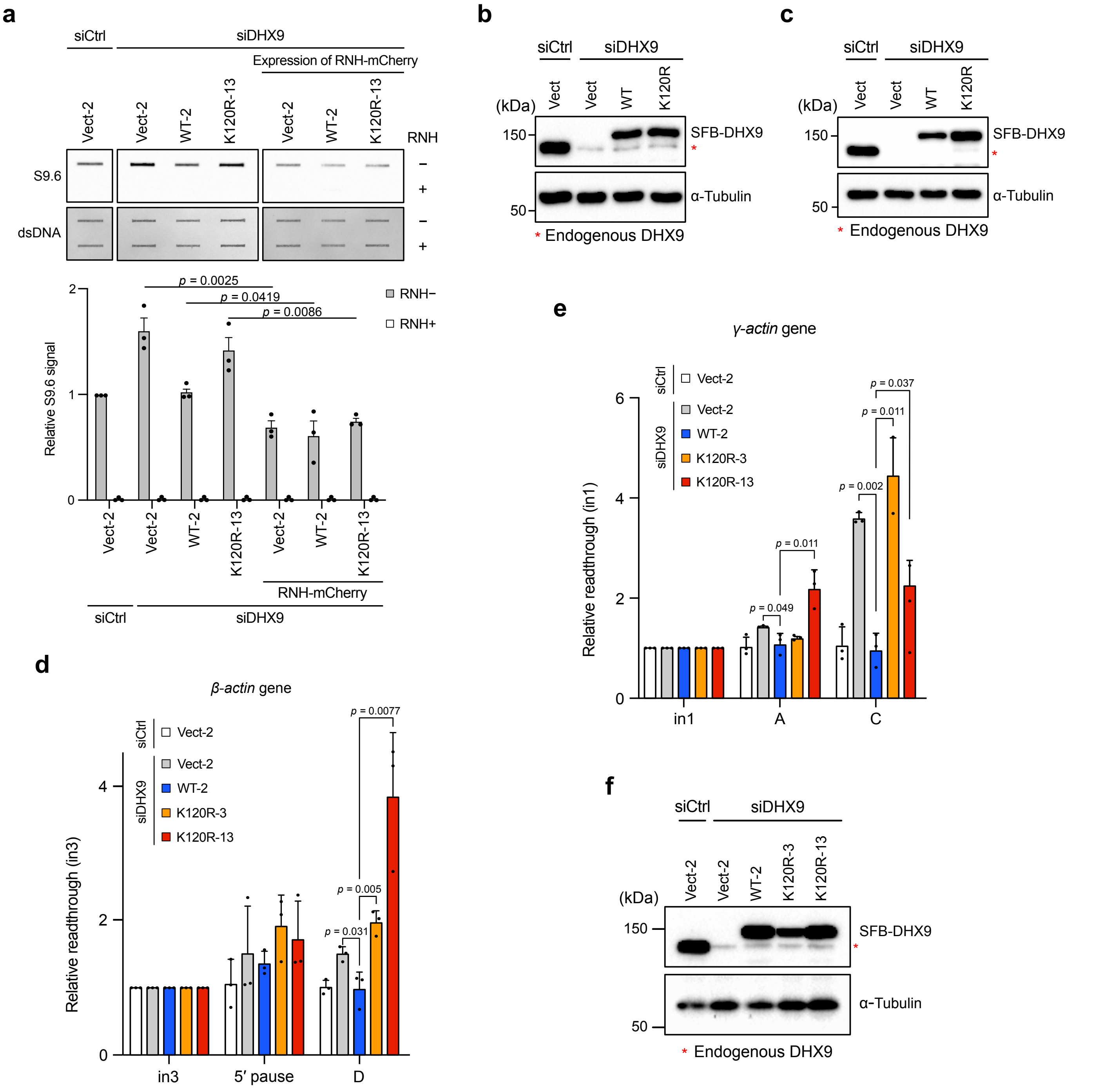

Supplementary Fig. 4 DHX9 SUMOylation suppresses R-loop accumulation and the impairment of transcription termination. **a** HeLa cells expressing DHX9^{K120R} displayed an accumulation of R-loops. HeLa derivative clones containing the vector, SFB-DHX9^{WT}, or SFB-DHX9^{K120R}, were transfected with siDHX9, followed by doxycycline induction. DNA/RNA hybrids isolated from nuclear extracts were treated with or without RNH and analyzed using a slot blot assay with the S9.6 antibody. The relative S9.6 signal was quantified (mean ± SEM with dots indicating the results of three independent experiments, two-sided *t*-test). Clones with additional transiently RNH-mCherry expression served as negative controls. Top: representative blots; Bottom: quantification of relative S9.6 signal. **b, c** Duplicated samples from Fig. 4c, d (**b**) and Fig. 4e, f (**c**) were used to test the protein levels of endogenous and SFB-tagged DHX9 by Western blot with anti-DHX9 antibody, three biological experiments. **d, e** Ablating K120 SUMOylation resulted in transcription termination deficiency. HeLa derivative clones containing the vector, SFB-DHX9^{WT}, or SFB-DHX9^{K120R}, were transfected with siDHX9, followed by doxycycline induction. Extracted nucleic acids were analyzed for read-through transcription of *β-actin* (**d**) and *γ-actin* (**e**) genes, respectively. Representative bar graph of two individual experiments. Values are normalized to *β-actin* in3 and *γ-actin* in1 and presented as mean ± SD, two-sided *t*-test. **f** Representative Western blot of three independent experiments shows the levels of SFB-DHX9^{WT} and SFB-DHX9^{K120R} in HeLa inducible clones used in experiments (**d**) and (**e**). Source data are provided as a Source Data file.

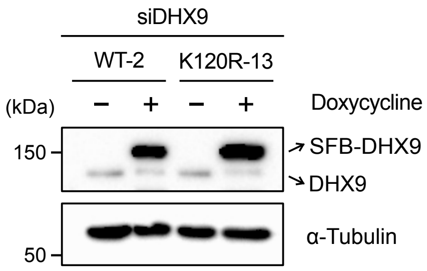

Supplementary Fig. 5 The vulnerability of DHX9^{K120R} cells to genotoxic stress can be rescued by RNH.
Levels of endogenous and SFB-tagged DHX9 proteins of inducible cell lines transfected with siDHX9 were confirmed by Western blot with anti-DHX9 antibody. Representative data of three independent experiments. Source data are provided as a Source Data file.

a

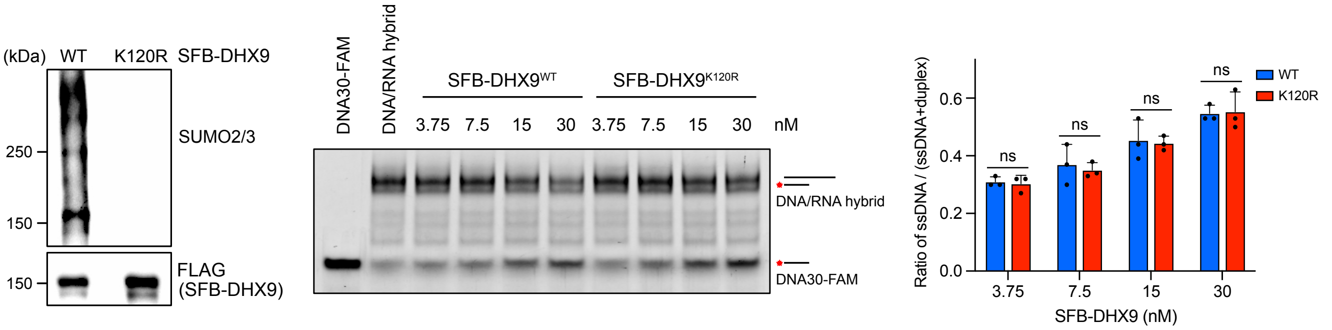

b

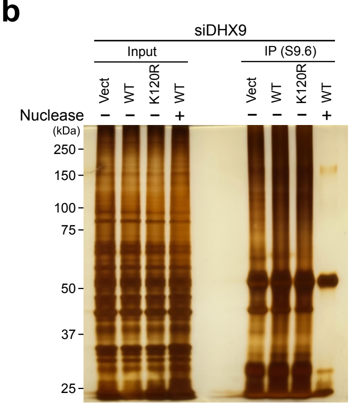

c

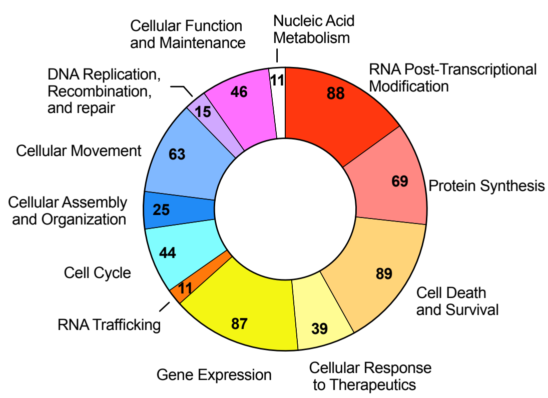

d

Gene Symbol	MW(kDa)	IgG # peptides	DHX9 # peptides
DHX9	140.87	4	308
DDX21	87.29	0	26
DHX30	133.85	0	23
HNRNPA2B1	37.41	0	20
PRPF8	273.43	0	20
DDX17	80.22	1	19
RBMX	42.31	1	19
DDX5	69.1	0	17
ADAR	135.98	0	12
PARP1	113.01	0	10
TOP2A	174.28	0	9
SFPQ	76.1	0	6
PRP19	55.15	0	5
SF3B1	145.74	0	5
H2AFX	15.14	0	3

e

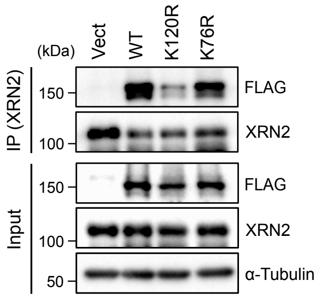

f

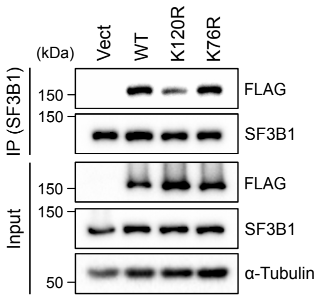

g

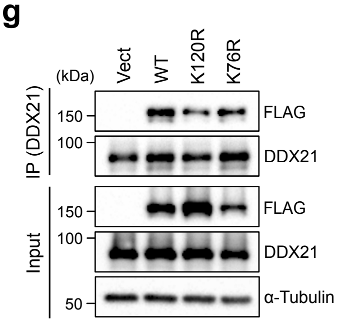

h

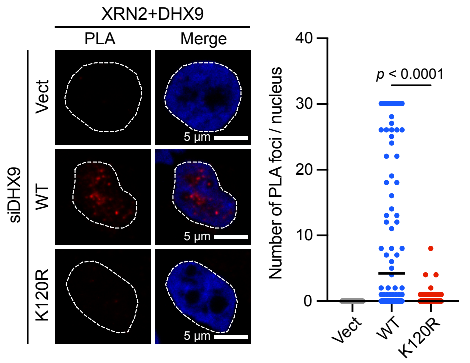

i

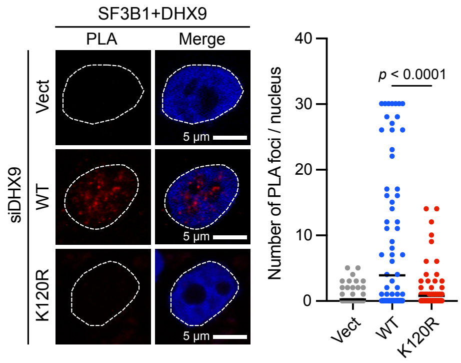

j

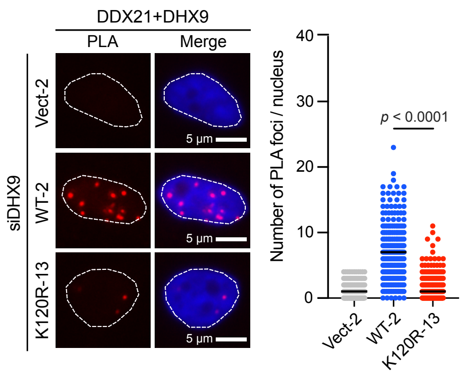

k

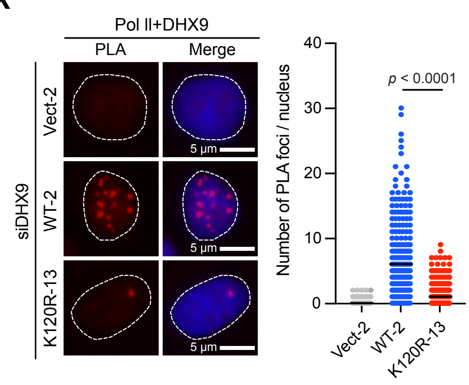

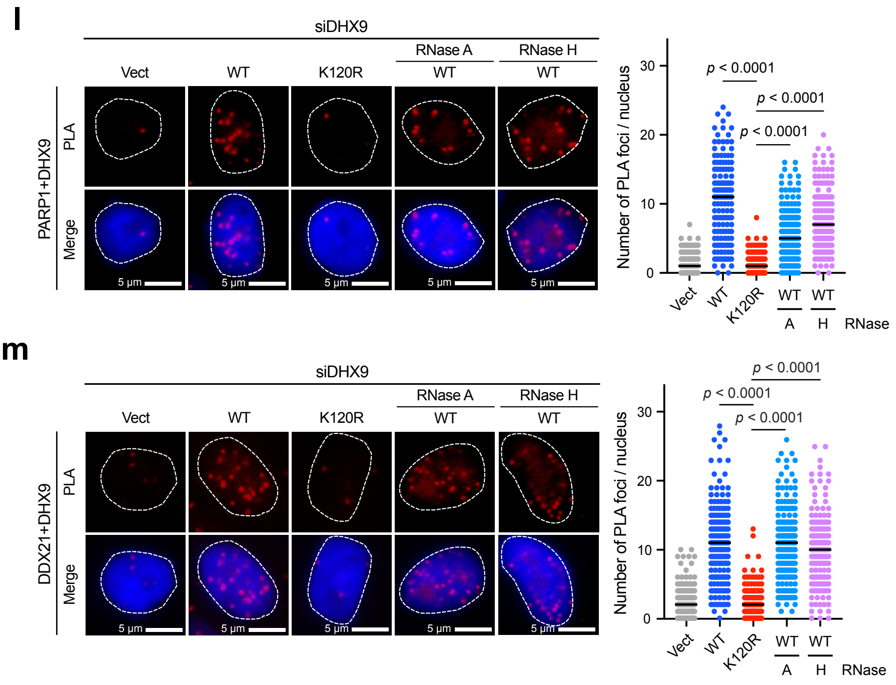

Supplementary Fig. 6 DHX9^{K120R} fails to efficiently associate with R-loops and R-loop interacting factors.
a Purified SFB-DHX9^{WT} and SFB-DHX9^{K120R} were used in the helicase assay with DNA30-FAM/RNA60 as the substrate. A representative Western blot (left) shows the SUMOylation of WT SFB-DHX9. The helicase activity of purified SFB-DHX9 variants was assessed by gel electrophoresis and fluorescence imaging (middle). The ratios of DNA30-FAM versus the total FAM signal in each group were quantified from three experiments, two-sided *t*-test (ns = no significance) (right). **b** Representative silver stain of DNA/RNA IP (n = 3) using the S9.6 antibody. Nuclear extracts from cells containing different SFB-DHX9 variants were treated with or without Universal Nuclease, followed by S9.6 IP. Samples were subjected to SDS-PAGE and silver staining. **c** Ingenuity Pathway Analysis assessed functional groups in 345 proteins detected by mass spectrometry (minimum 2 peptides). A donut chart illustrates the number of proteins grouped by specific functions. **d** A table summarizes R-loop interacting proteins co-purified with DHX9 affinity purification. **e**-**g** IP of endogenous XRN2 (**e**), SF3B1 (**f**), and DDX21 (**g**) using HeLa cell lysates containing either the vector or different SFB-DHX9 variants. The interactions between immunoprecipitated proteins and SFB-DHX9 variants were determined by Western blot for at least two times. **h**-**k** The association of DHX9 variants with XRN2 (**h**), SF3B1 (**i**), DDX21 (**j**), and Pol II (**k**) in DHX9-depleted HeLa cells carrying the vector, SFB-DHX9^{WT}, or SFB-DHX9^{K120R} was assessed by PLA with the indicated antibodies. Analyses for **h** and **i** were performed with transient expression of SFB-DHX9 variants, while those for **j** and **k** were conducted in HeLa inducible lines. **l**, **m** The association of SFB-DHX9 variants with PARP1 (**l**) or DDX21 (**m**) was analyzed by PLA. PFA-fixed samples were further treated with or without RNase A or RNase H before incubating with primary antibodies. For all PLA analyses (h-m) (red), 150 nuclei under each condition were counted and analyzed by two-sided Mann-Whitney *U* test. The black line indicates the median. Source data are provided as a Source Data file.

a

SUMO-interacting motif (SIM) prediciton by GPS-SUMO

	ID	Position	Peptide	Score	Cut-off	Type	Source
SIM1	PARP1	801-805	YEKLKTDIKVVDRDSEEAE	0.9439	0.5	SUMO interaction	Pred.
	PARP1	916-920	HTSQGDPIGLILLGEVALG	0.7503	0.5	SUMO interaction	Pred.
	PARP1	990-994	SLLYNEYIVYDIAQVNLKY	0.7097	0.5	SUMO interaction	Pred.
SIM2	PARP1	831-835	TTHNAYDLEVIDIFKIERE	0.7039	0.5	SUMO interaction	Pred.
	PARP1	592-596	IFRSWGRVGTVIGSNKLEQ	0.6902	0.5	SUMO interaction	Pred.

b

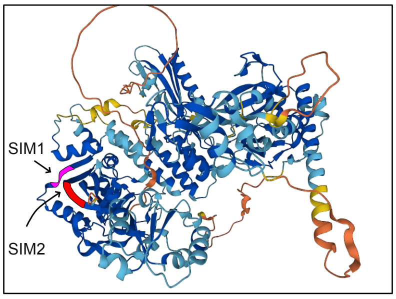

c

PARP1

```
                             SIM1                                SIM2
Homo sapiens        VNYEKLKTDIKVVDRDSEEAEIIRKYVKNTHATTHNAYDLEVIDIFKIEREG
Mus musculus        VNYEKLKTDIKVVDRDSEEAEVIRKYVKNTHATTHNAYDLEVIDIFKIEREG
Rattus norvegicus   VNYEKLKTDIKVVDRDSEEAEVIRKYVKNTHATTHNAYDLEVIDIFKIEREG
Bos taurus          VNYEKLKTDIKVVDKDSEEAEIIRKYVKNTHATTHNAYDLEVVDIFKIEREG
Danio rerio         INYEKLKTKIEVVDKSSHEAQLILQYVKNTHAATHNTYTLDVEEIFKIEREG
```

DDX21

```
Homo sapiens        VFNVAKKYMKSTYEQVDLIGKKTQKTAITVEHLA
Mus musculus        VFNVAKKYMKSTYEQVDLIGKKTQKAAITVEHLA
Rattus norvegicus   VFNVAKKYMKSTYEQVDLIGKKTQKAAITVEHLA
Bos taurus          VFNVAKKYMKSTYEQVDLIGKKTQKTAITVEHLA
Danio rerio         VYDVAKKYMRSQFIHVDLIGKKTQKAATTVEHLA
```

d

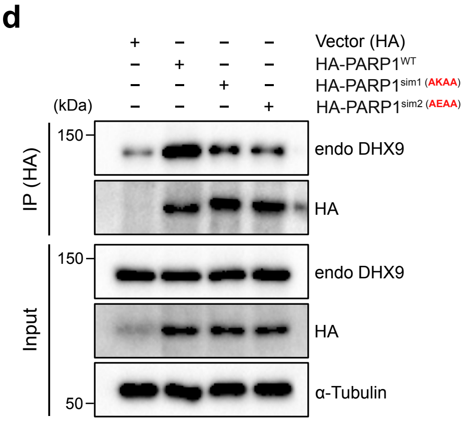

Supplementary Fig. 7 DHX9 SUMOylation enhances its association with PARP1 and DDX21 via SUMO-SIM interaction. **a** Potential SIMs of PARP1 predicted by GPS-SUMO 6.0. SIM1 and SIM2 examined in this study were highlighted in red. **b** The positions of SIM1 and SIM2 on the PARP1 structure were marked in magenta and red, respectively. The predicted PAPR1 structure was generated by AlphaFold. **c** Protein sequence alignment of PARP1 and DDX21 across various vertebrates reveals conserved SIMs highlighted in red. **d** HeLa cells were transfected with either the empty vector or different HA-PAPR1 variants for 2 d, and then collected for IP using anti-HA beads. The interaction between HA-PARP1 variants and endogenous DHX9 was determined by Western blot for at least two times. Source data are provided as a Source Data file.

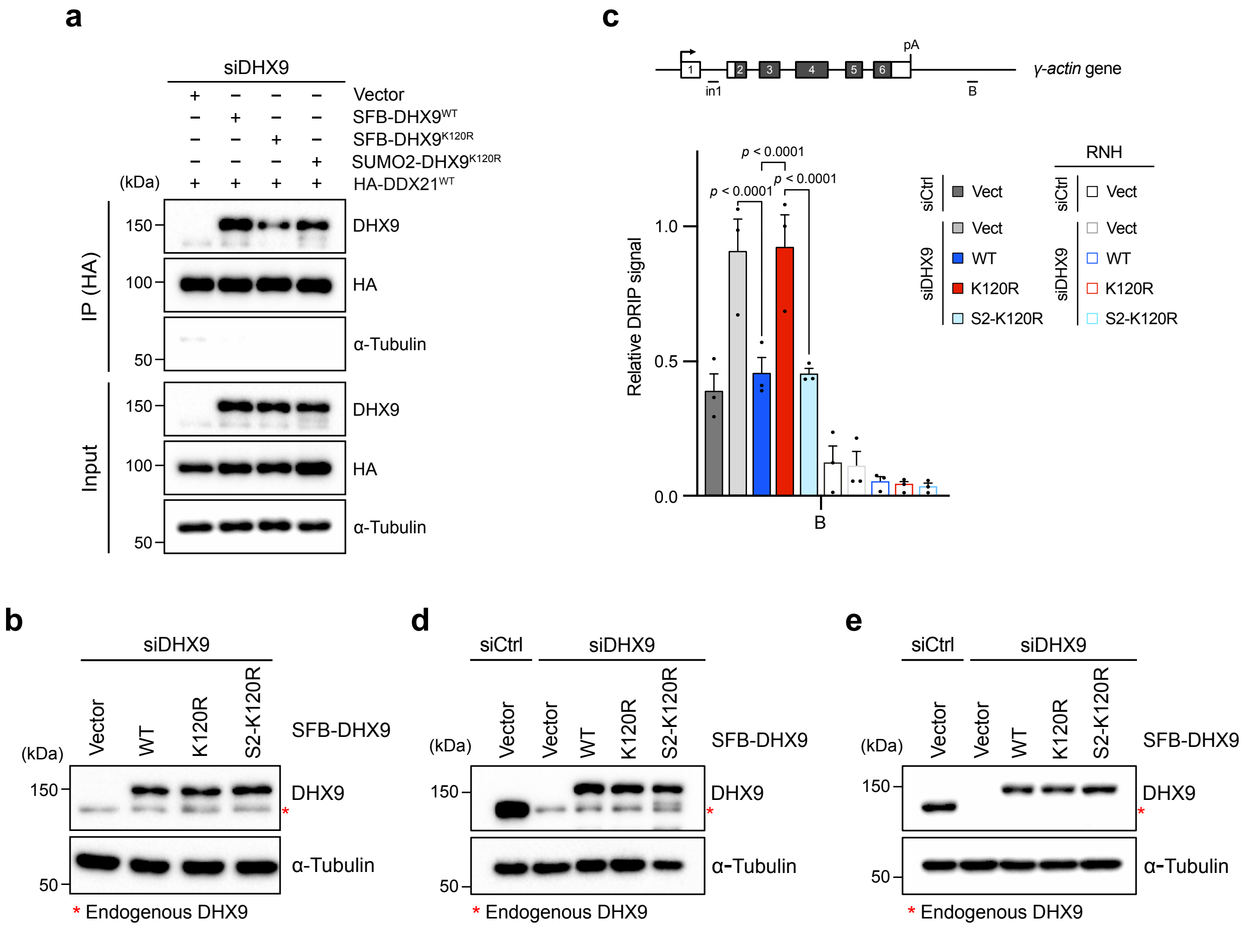

Supplementary Fig. 8 Fusion of a SUMO2 to DHX9^{K120R} bypasses the SUMOylation of DHX9.
a The interaction between SFB-DHX9 variants and HA-DDX21 was assessed by co-IP. HeLa cells, transfected with siDHX9, were co-transfected with indicated constructs for 2 d. Cells were collected for IP using anti-HA beads. Levels of SFB-DHX9 variants and SUMO2-DHX9^{K120R} co-precipitated by HA-DDX21 were determined by Western blot with the indicated antibodies. **b** Duplicated samples from Fig. 8b were used to examine the protein level of SFB-DHX9 variants and SUMO2-DHX9^{K120R} by Western blot with anti-DHX9 antibody. **c** The relative R-loop level at the B region of *γ-actin* gene was determined by RT-qPCR. The representative bar graph was from three separate experiments. Values are normalized to *γ-actin* in1 and presented as mean ± SEM (two-sided *t*-test). **d** Duplicated samples from Fig. 8c and Supplementary Fig. 8c were used to confirm the protein level of SFB-DHX9 variants and SUMO2-DHX9^{K120R} by Western blot with anti-DHX9 antibody. **e** Duplicated samples from Fig. 8d were used to confirm the protein level of SFB-DHX9 variants and SUMO2-DHX9^{K120R} by Western blot with anti-DHX9 antibody. Western blots for validating knockdown of DHX9 and the expression of DHX9 variants were repeated at least three times. Source data are provided as a Source Data file.

Supplementary Table 1

The information of RNAi used in this study

siRNA (target gene)	Sequences	Identifier	Company
DHX9	GAGUGUAACAUCGUAGUAAdTdT	s4019	ThermoFisher
UBC9	GGAAUACAGGAACUUCUAAdTdT	s14590	ThermoFisher
Negative Control	N/A	4390843	ThermoFisher

Supplementary Table 2

Small molecule inhibitors used in this study

Name	Soure	Identifier
Berzosertib	Selleckchem	S7102
Camptothecin	Selleckchem	S1288
Cisplatin	Selleckchem	S1166
Hydroxyurea	Sigma-Aldrich	H8627

Supplementary Table 3

Primers used for cloning of DHX9, PARP1, DDX21, and site-directed mutagenesis

Primer name	Primers (5'-3')
DHX9 Nested PCR primers-Forward	GAGTCACACACGGTCCTAAG
DHX9 Nested PCR primers-Reverse	AACAAACAAACTACACGGCA
DHX9 entry clone primers-Forward	CACCATGGGTGACGTTAAAAATTTTCTG
DHX9 entry clone primers-Reverse	TTAATAGCCGCCACCTCCTCTTC
DHX9 siRNA resistant-Forward	GTCGTAACTCAGCCCAGAAGAATC
DHX9 siRNA resistant-Reverse	AATATTACACTCTGCTGCTCGG
DHX9_K76R-Forward	AAATGAAATAAGGAGTGAAGAAGTTC
DHX9_K76R-Reverse	ATTCGAACCAAATAGTTAAC
DHX9-K120R-Forward	CTGGCTCTCAGAGCAGAAAATAATTC
DHX9-K120R-Reverse	ATGTGGAGGAAGAGGTCC
NheI-SFB-DHX9-Forward	ATCCGCTAGCGCTACCGGA
AgeI-SFB-DHX9-Reverse	TAACCGGTTTAATAGCCGCCACCTCCTC
DDX21_For	CACCATGCCGGGAAAACTCCGT
DDX21_Rev	CCTTTTATTGACCAAATGCTTTACT
PARP1_For	CACCATGGCGGAGTCTTCGGAT
PARP1_Rev	CTTTTACCACAGGGAGGTCTTA
PARP1_sim1_F1	GCAGCCGACAGAGATTCTGAAGAAG
PARP1_sim1_R1	CTTGGCGTCAGTTTTGAGCTTCTC
PARP1_sim2_F1	GCCGCCGATATCTTTAAGATAGAGCGTG
PARP1_sim2_R1	TTCTGCGTCATACGCATTGTGTGTG
AgeI-EXP-D9_F	GGTACAAGTTTGTACAAAAAAGCAG
AgeI-EXP-D9_R	GGTTTCTTTCATGGTGGCCTC
AgeI-S2(12)-F	GAACCGGTACTGAGAACAACGATCATATTA
AgeI-S2(91)-R	TATACCGGTCGTCTGCTGTTGGAACAC
DDX21_SIM1_For	GCGGCTGGTAAAAAGACTCAGAAAAC
DDX21_SIM1_Rev	GGCCGCCTGTTCATATGTAGATTTCATG
A121G_For	GCTCTCAAAGGTGAAAATAATTCTGAGG
A121G_Rev	CAGATGTGGAGGAAGAG
E122A_For	CTCAAAGCAGCAAATAATTCTGAG
E122A_Rev	AGCCAGATGTGGAGG
HIS-GST-D9_1-399For	TAAGCGGCCGCATAATG
HIS-GST-D9_1-399Rev	TTCCAGAATCTCACTTTCAAATTTC

Supplementary Table 4

Primers used in this study for DRIP-RT-qPCR analysis and the read-through assay of transcriptional termination

Primer names	Sequences (5'-3')
β-actin_in 1_Forward	CGGGGTCTTTGTCTGAGC
β-actin_in 1_Reverse	CAGTTAGCGCCCAAAGGAC
β-actin_in 3_Forward	TAACACTGGCTCGTGTGACAA
β-actin_in 3_Reverse	AAGTGCAAAGAACACGGCTAA
β-actin_5' pause_Forward	TTACCCAGAGTGCAGGTGTG
β-actin_5' pause_Reverse	CCCCAATAAGCAGGAACAGA
β-actin_pause_Forward	GGGACTATTTGGGGGTGTCT
β-actin_pause_Reverse	TCCCATAGGTGAAGGCAAAG
β-actin_C_Forward	TGGGCCACTTAATCATTCAAC
β-actin_C_Reverse	CCTCACTTCCAGACTGACAGC
β-actin_D_Forward	CAGTGGTGTGGTGTGATCTTG
β-actin_D_Reverse	GGCAAAACCCTGTATCTGTGA
γ-actin_in 1_Forward	CCGCAGTGCAGACTTCCGAG
γ-actin_in 1_Reverse	CGGGCGCGTCTGTAACACGG
γ-actin_A_Forward	TTCGTGGGCTGGTGAGAAAA
γ-actin_A_Reverse	CTCCAACACCCAAACCCACT
γ-actin_B_Forward	GGGTCAAGGGATCGTTCTG
γ-actin_B_Reverse	GCCTGGAGCTCAGTAAGC
γ-actin_C_Forward	GAGGTTTGAGACTGCAGTGAG
γ-actin_C_Reverse	CAGACATAATTTTGTGGGGTTTG

Supplementary Table 5

Primers used in this study for helicase assay

Primer names	Sequences (5'-3')
DNA30-FAM	CGAATTCGAGCTCGCCCGGGGATCCTCTAG-FAM
OligoRNA60-T7	GAAATTAATACGACTCACTATAGGGAGACCCGAATTCGAGCTCGC CCGGGGATCCTCTAGtatagtgagtcgtatta

Supplementary methods

Protein Sequence Analysis by LC-MS/MS

The samples were prepared and analyzed at the Taplin Biological Mass Spectrometry Facility, Harvard Medical School. The methods were kindly provided by Ross Tomaino, Associate Director of the Taplin Biological Mass Spectrometry Facility.

Excised gel bands were subjected to a modified in-gel trypsin digestion procedure (1). Gel pieces were washed and dehydrated with acetonitrile for 10 min. followed by removal of acetonitrile. Pieces were then completely dried in a speed-vac. Rehydration of the gel pieces was with 50 mM ammonium bicarbonate solution containing 12.5 ng/µl modified sequencing-grade trypsin (Promega) at 4 ºC. After 45 min, the trypsin solution was removed and replaced with 50 mM ammonium bicarbonate solution. Samples were then placed in a 37ºC room overnight. Peptides were extracted by removing the ammonium bicarbonate solution, followed by one wash containing 50% acetonitrile and 1% formic acid. The extracts were then dried in a speed-vac for 1 h. The samples were then stored at 4ºC until analysis.

On the day of analysis, the samples were reconstituted in 5 - 10 µl of HPLC solvent A (2.5% acetonitrile, 0.1% formic acid). A nano-scale reverse-phase HPLC capillary column was created by packing 2.6 µm C18 spherical silica beads into a fused silica capillary (100 µm inner diameter x ~30 cm length) with a flame-drawn tip (2). After equilibrating the column, each sample was loaded via a Famos auto sampler (LC Packings) onto the column. A gradient was formed, and peptides were eluted with increasing concentrations of solvent B (97.5% acetonitrile, 0.1% formic acid).

As peptides eluted, they were subjected to electrospray ionization and then entered into a Velos Orbitrap Elite ion-trap mass spectrometer (Thermo Fisher Scientific). Peptides were detected, isolated, and fragmented to produce a tandem mass spectrum of specific fragment ions for each peptide. Peptide sequences (and hence protein identity) were determined by matching protein databases with the acquired fragmentation pattern by the software program, Sequest (ver. 28, rev. 13, Thermo Fisher Scientific) (3). The protein database was downloaded from Uniprot on June 20th, 2017. The peptide false discovery rate (FDR) for IgG IP sample was 0.6% and for DHX9 IP sample was 1.05%. The database was indexed for fully tryptic and allowing for up to two missed cleavages.

References:

1. *Shevchenko, et al., Anal Chem. 68:850-858, 1996*
2. *Peng, J and Gygi S.P. Proteomics: The move to mixtures, J. Mass Spec. Oct:36(10):1083-91*
3. *Eng. et al., J. Am. Soc. Mass. Spectrom. 5:976-989, 1994*
