# Supplementary material for: DHX9 SUMOylation is required for the suppression of R-loop-associated genome instability
Source: Nat Commun. 2024 Jul 17;15:6009. doi: 10.1038/s41467-024-50428-4 (PMC11255299; doi:10.1038/s41467-024-50428-4)
Supplement: Supplementary file 3 — Reporting Summary [file 41467_2024_50428_MOESM3_ESM.pdf]

nature portfolio

Corresponding author(s): Ching-Shyi Peter Wu

Last updated by author(s): Jun 11, 2024

Reporting Summary

Nature Portfolio wishes to improve the reproducibility of the work that we publish. This form provides structure for consistency and transparency in reporting. For further information on Nature Portfolio policies, see our Editorial Policies and the Editorial Policy Checklist.

Statistics

For all statistical analyses, confirm that the following items are present in the figure legend, table legend, main text, or Methods section.

n/a	Confirmed	
☐	☒	The exact sample size (*n*) for each experimental group/condition, given as a discrete number and unit of measurement
☐	☒	A statement on whether measurements were taken from distinct samples or whether the same sample was measured repeatedly
☐	☒	The statistical test(s) used AND whether they are one- or two-sided<br>*Only common tests should be described solely by name; describe more complex techniques in the Methods section.*
☒	☐	A description of all covariates tested
☒	☐	A description of any assumptions or corrections, such as tests of normality and adjustment for multiple comparisons
☐	☒	A full description of the statistical parameters including central tendency (e.g. means) or other basic estimates (e.g. regression coefficient) AND variation (e.g. standard deviation) or associated estimates of uncertainty (e.g. confidence intervals)
☐	☒	For null hypothesis testing, the test statistic (e.g. *F*, *t*, *r*) with confidence intervals, effect sizes, degrees of freedom and *P* value noted<br>*Give P values as exact values whenever suitable.*
☒	☐	For Bayesian analysis, information on the choice of priors and Markov chain Monte Carlo settings
☒	☐	For hierarchical and complex designs, identification of the appropriate level for tests and full reporting of outcomes
☒	☐	Estimates of effect sizes (e.g. Cohen's *d*, Pearson's *r*), indicating how they were calculated

Our web collection on statistics for biologists contains articles on many of the points above.

Software and code

Policy information about availability of computer code

Data collection	Western blot data: ChemiDoc MP (Bio-Rad),<br>Immunofluorescence images: EVOS M7000 (ThermoFisher)<br>Real Time-PCR analysis: CFX DUET qPCR (Bio-Rad)<br>Cell Titer-Glo 2.0 luminescent viability assay: ELISA reader (Biotek)<br>Images of helicase assay: Typhoon biomolecular imager (cytiva)<br>Flow-cytometric data: LSRFortessa Flow cytometer (BD Bioscience)
Data analysis	Immunofluorescence images: Celleste V4.1.1, ThermoFisher.<br>Western blot and helicase assay data: ImageLab V6.1.0, Bio-Rad.<br>Flow cytometry data: Kaluza 2.2.1, Beckman Coulter.<br>Cell Titer-Glo 2.0 luminescent viability assay: Gen 5 V3.11, Biotek.<br>RT-qPCR data: Bio-Rad CFX V4.1.2433.<br>Statistics: GraphPad Prism 9, GraphPad Software, Inc..

For manuscripts utilizing custom algorithms or software that are central to the research but not yet described in published literature, software must be made available to editors and reviewers. We strongly encourage code deposition in a community repository (e.g. GitHub). See the Nature Portfolio guidelines for submitting code & software for further information.

Data

Policy information about availability of data

All manuscripts must include a data availability statement. This statement should provide the following information, where applicable:

- Accession codes, unique identifiers, or web links for publicly available datasets
- A description of any restrictions on data availability
- For clinical datasets or third party data, please ensure that the statement adheres to our policy

All the supporting data for this study can be found in the manuscript and its supplementary documents. The Uniprot (https://www.uniprot.org/), GPS-SUMO (https://sumo.biocuckoo.cn/), SUMOplot (https://www.abcepta.com/sumoplot), and AlphaFold (https://alphafold.ebi.ac.uk/) databases are publicly accessible. The data of DHX9 interactome are available via ProteomeXchange with the identifier PXD044366 (https://www.ebi.ac.uk/pride/archive/projects/PXD044366). Source data are provided with this paper.

Research involving human participants, their data, or biological material

Policy information about studies with human participants or human data. See also policy information about sex, gender (identity/presentation), and sexual orientation and race, ethnicity and racism.

Reporting on sex and gender	*Use the terms sex (biological attribute) and gender (shaped by social and cultural circumstances) carefully in order to avoid confusing both terms. Indicate if findings apply to only one sex or gender; describe whether sex and gender were considered in study design; whether sex and/or gender was determined based on self-reporting or assigned and methods used. Provide in the source data disaggregated sex and gender data, where this information has been collected, and if consent has been obtained for sharing of individual-level data; provide overall numbers in this Reporting Summary. Please state if this information has not been collected. Report sex- and gender-based analyses where performed, justify reasons for lack of sex- and gender-based analysis.*
Reporting on race, ethnicity, or other socially relevant groupings	*Please specify the socially constructed or socially relevant categorization variable(s) used in your manuscript and explain why they were used. Please note that such variables should not be used as proxies for other socially constructed/relevant variables (for example, race or ethnicity should not be used as a proxy for socioeconomic status). Provide clear definitions of the relevant terms used, how they were provided (by the participants/respondents, the researchers, or third parties), and the method(s) used to classify people into the different categories (e.g. self-report, census or administrative data, social media data, etc.) Please provide details about how you controlled for confounding variables in your analyses.*
Population characteristics	*Describe the covariate-relevant population characteristics of the human research participants (e.g. age, genotypic information, past and current diagnosis and treatment categories). If you filled out the behavioural & social sciences study design questions and have nothing to add here, write "See above."*
Recruitment	*Describe how participants were recruited. Outline any potential self-selection bias or other biases that may be present and how these are likely to impact results.*
Ethics oversight	*Identify the organization(s) that approved the study protocol.*

Note that full information on the approval of the study protocol must also be provided in the manuscript.

Field-specific reporting

Please select the one below that is the best fit for your research. If you are not sure, read the appropriate sections before making your selection.

☒ Life sciences ☐ Behavioural & social sciences ☐ Ecological, evolutionary & environmental sciences

For a reference copy of the document with all sections, see nature.com/documents/nr-reporting-summary-flat.pdf

Life sciences study design

All studies must disclose on these points even when the disclosure is negative.

Sample size	The sample size of each experiment was based on the experimental methods or follow the standard experimental design used in published studies.
Data exclusions	Not applied.
Replication	Experiments were repeated independently three times, unless otherwise indicated in the figure legends.
Randomization	All cells used in experiments were randomly allocated into groups.
Blinding	The investigators were blinded to experimental conditions before data analysis.

Reporting for specific materials, systems and methods

We require information from authors about some types of materials, experimental systems and methods used in many studies. Here, indicate whether each material, system or method listed is relevant to your study. If you are not sure if a list item applies to your research, read the appropriate section before selecting a response.

Materials & experimental systems

n/a	Involved in the study
☐	☒ Antibodies
☐	☒ Eukaryotic cell lines
☒	☐ Palaeontology and archaeology
☒	☐ Animals and other organisms
☒	☐ Clinical data
☒	☐ Dual use research of concern
☒	☐ Plants

Methods

n/a	Involved in the study
☒	☐ ChIP-seq
☐	☒ Flow cytometry
☒	☐ MRI-based neuroimaging

Antibodies

Antibodies used

Anti-SUMO1 (Cell Signaling Technology, #4930, 1:1000) for IB;
Anti-NONO (Cell Signaling Technology, #90336, 1:1000) for IB;
Anti-Ubc9 (Cell Signaling Technology, #4786, 1:1000) for IB;
Anti-DDX21 (Santa Cruz, sc-376953, 1:500) for IB;
Anti-DDX21 (Santa Cruz, sc-376953, 1:100) for PLA;
Anti-PRP19 (Santa Cruz, sc-514338, 1:500) for IB;
Anti-Pol ll (Santa Cruz, sc-47701, 1:400) for IB;
Anti-Pol ll (Santa Cruz, sc-47701, 1:100) for PLA;
Anti-PARP1 (Santa Cruz, sc-74470, 1:400) for IB;
Anti-PARP1 (Santa Cruz, sc-8007, 1:400) for IB;
Anti-PARP1 (Santa Cruz, sc-8007, 1:100) for PLA;
Anti-ADAR1 (Santa Cruz, sc-73408, 1:500) for IB;
Anti-DsRed (Santa Cruz, sc-390909, 1:500) for IB;
Anti-SF3B1 (Santa Cruz, sc-514655, 1:500) for IB;
Anti-SF3B1 (Santa Cruz, sc-514655, 1:100) for PLA;
Anti-XRN2 (Santa Cruz, sc-365258, 1:400) for IB;
Anti-XRN2 (Santa Cruz, sc-365258, 1:100) for PLA;
Anti-DHX9 (Abcam, ab26271, 1:2000) for IB;
Anti-DHX9 (Abcam, ab26271, 1:400) for PLA;
Anti-SUMO2/3 (Abcam, ab3742, 1:1000) for IB;
Anti-SUMO2/3 (Abcam, ab3742, 1:600) for PLA;
Anti-HA Tag (ThermoFisher, 715500, 1:2000) for IB;
Anti-RPA32 pS33 (Bethyl Lab, A300-246A, 1:2000) for IB;
Anti-phospho-Histone H2A.X (S139) (Millipore, 05-636, 1:250) for IF;
Anti-FLAG (Merck, F7425, 1:2000) for IB;
Anti-FLAG (Merck, F7425, 1:500) for IF;
Anti-FLAG (Merck, F7425, 1:500) for PLA;
Anti-S9.6 (Merck, MABE1095, 1:300) for PLA;
Anti-S9.6 (Merck, MABE1095, 1:2000) for SB;
Anti-alpha-Tubulin (Merck, T6074, 1:12000) for IB;
Anti-mouse-HRP (ThermoFisher, G21040, 1:10000) for IB;
Anti-rabbit-HRP (ThermoFisher, G21234, 1:10000) for IB;
Anti-mouse Alexa Fluor-594 (ThermoFisher, A-11005, 1:400) for IF;
Anti-rabbit Alexa Fluor-488 (ThermoFisher, A-11034, 1:400) for IF.

Validation

Anti-SUMO1 for IB, https://www.cellsignal.com/products/primary-antibodies/sumo-1-antibody/4930
Anti-NONO for IB, https://www.cellsignal.com/products/primary-antibodies/nono-antibody/90336
Anti-Ubc9 for IB, https://www.cellsignal.com/products/primary-antibodies/ubc9-d26f2-xp-rabbit-mab/4786
Anti-DDX21 for IB and PLA, https://www.scbt.com/p/ddx21-antibody-d-8;
Anti-PRP19 for IB, https://www.scbt.com/p/prp19-antibody-g-7;
Anti-Pol ll for IB and PLA, https://www.scbt.com/p/pol-ii-antibody-ctd4h8;
Anti-PARP1 for IB, https://www.scbt.com/p/parp-1-antibody-b-10;
Anti-PARP1 for IB and PLA, https://www.scbt.com/p/parp-1-antibody-f-2;
Anti-ADAR1 for IB, https://www.scbt.com/p/adar1-antibody-15-8-6
Anti-DsRed for IB, https://www.scbt.com/p/dsred-antibody-e-8
Anti-SF3B1 for IB and PLA, https://www.scbt.com/p/sap-155-antibody-b-3;
Anti-XRN2 for IB and PLA, https://www.scbt.com/p/xrn2-antibody-h-3;
Anti-RPA32 pS33 for IB, https://www.fortislife.com/products/primary-antibodies/rabbit-anti-phospho-rpa32-s33-antibody/BETHYL-A300-246
Anti-DHX9 for IB and PLA, https://www.abcam.com/products/primary-antibodies/rna-helicase-a-antibody-ab26271.html;

Anti-SUMO2/3 for IB and PLA, https://www.abcam.com/products/primary-antibodies/sumo-2--sumo-3-antibody-ab3742.html;
Anti-HA Tag for IB, https://www.thermofisher.com/antibody/product/HA-Tag-Antibody-clone-SG77-Polyclonal/71-5500;
Anti-phospho-Histone H2A.X (S139) for IF, https://www.merckmillipore.com/TW/zh/product/Anti-phospho-Histone-H2A.X-Ser139-Antibody-clone-JBW301,MM_NF-05-636;
Anti-FLAG for IB, IF and PLA,
https://www.sigmaaldrich.com/TW/en/product/sigma/f7425?gclid=Cj0KCQjwmICoBhDxARIsABXkXlJVtm1akqJl4xWUU_NyQXHxIY03aggOjDzElhxLi6eNSRpK1-g-vW8aAh-jEALw_wcB;
Anti-S9.6 for PLA and SB, https://www.merckmillipore.com/TW/zh/product/Anti-DNA-RNA-Hybrid-Antibody-clone-S9.6,MM_NF-MABE1095
Anti-alpha-Tubulin for IB, https://www.sigmaaldrich.com/TW/en/product/sigma/t6074?gclid=Cj0KCQjwmICoBhDxARIsABXkXlKB_qSdon8-L5piRUFN0XKftTZa6GGkSqci2vsaclsNmxqnA0ZOnwoaAik9EALw_wcB.
Anti-mouse-HRP for IB, https://www.thermofisher.cn/cn/zh/antibody/product/Goat-anti-Mouse-IgG-H-L-Cross-Adsorbed-Secondary-Antibody-Polyclonal/G-21040?adobe_mc=MCMID%7C68100346614437537653070856881765654608%7CMCAID%3D30C110F1244C2E29-60000F42A2662576%7CMCORGID%3D5B135A0C5370E6B40A490D44%40AdobeOrg%7CTS=1614293705
Anti-rabbit-HRP for IB, https://www.thermofisher.cn/cn/zh/antibody/product/Goat-anti-Rabbit-IgG-H-L-Cross-Adsorbed-Secondary-Antibody-Polyclonal/G-21234?adobe_mc=MCMID%7C68100346614437537653070856881765654608%7CMCAID%3D30C110F1244C2E29-60000F42A2662576%7CMCORGID%3D5B135A0C5370E6B40A490D44%40AdobeOrg%7CTS=1614293705
Anti-mouse Alexa Fluor-594 for IF, https://www.thermofisher.cn/cn/zh/antibody/product/Goat-anti-Mouse-IgG-H-L-Cross-Adsorbed-Secondary-Antibody-Polyclonal/A-11005?adobe_mc=MCMID%7C68100346614437537653070856881765654608%7CMCAID%3D30C110F1244C2E29-60000F42A2662576%7CMCORGID%3D5B135A0C5370E6B40A490D44%40AdobeOrg%7CTS=1614293705
Anti-rabbit Alexa Fluor-488 for IF, https://www.thermofisher.cn/cn/zh/antibody/product/Goat-anti-Rabbit-IgG-H-L-Highly-Cross-Adsorbed-Secondary-Antibody-Polyclonal/A-11034?adobe_mc=MCMID%7C68100346614437537653070856881765654608%7CMCAID%3D30C110F1244C2E29-60000F42A2662576%7CMCORGID%3D5B135A0C5370E6B40A490D44@AdobeOrg%7CTS%3D1614293705

Eukaryotic cell lines

Policy information about cell lines and Sex and Gender in Research

Cell line source(s)	HeLa and U2OS cancer cell lines were purchased directly from the ATCC.
Authentication	The cell line obtained from a commercial source, and was not authenticated further.
Mycoplasma contamination	All cell lines were confirmed without mycoplasma contamination.
Commonly misidentified lines (See ICLAC register)	N/A

Plants

Seed stocks	*Report on the source of all seed stocks or other plant material used. If applicable, state the seed stock centre and catalogue number. If plant specimens were collected from the field, describe the collection location, date and sampling procedures.*
Novel plant genotypes	*Describe the methods by which all novel plant genotypes were produced. This includes those generated by transgenic approaches, gene editing, chemical/radiation-based mutagenesis and hybridization. For transgenic lines, describe the transformation method, the number of independent lines analyzed and the generation upon which experiments were performed. For gene-edited lines, describe the editor used, the endogenous sequence targeted for editing, the targeting guide RNA sequence (if applicable) and how the editor was applied.*
Authentication	*Describe any authentication procedures for each seed stock used or novel genotype generated. Describe any experiments used to assess the effect of a mutation and, where applicable, how potential secondary effects (e.g. second site T-DNA insertions, mosiacism, off-target gene editing) were examined.*

Flow Cytometry

Plots

Confirm that:

☒ The axis labels state the marker and fluorochrome used (e.g. CD4-FITC).

☒ The axis scales are clearly visible. Include numbers along axes only for bottom left plot of group (a 'group' is an analysis of identical markers).

☒ All plots are contour plots with outliers or pseudocolor plots.

☒ A numerical value for number of cells or percentage (with statistics) is provided.

Methodology

Sample preparation	To monitor cell death, HeLa derivative clones were transfected with control or DHX9 siRNA for 24 h before inducing SFB-DHX9 proteins by doxycycline (200 ng/ml). 64 h after siRNA transfection, floating and attached cells were collected for the Annexin V-APC analysis (Biolegend) according to the manufacturer's instructions. To monitor the correlation of accumulated γH2AX signal and the overexpression of RNH, we used flow cytometry to determine the double-positive signal of γH2AX and RNH. HeLa derivative clones were transfected with control or DHX9 siRNA for 24 h, followed by the transfection of RNH-

	mCherry or the empty vector with doxycycline added simultaneously. 64 h after siRNA transfection, cells were collected and fixed, stained with anti-γH2AX, washed, and incubated with Alexa-488-conjugated secondary antibody.
Instrument	All data were acquired using the LSRFortessa Flow cytometer (BD Bioscience)
Software	Kaluza software V2.2.1 (Beckman Coulter)
Cell population abundance	No sorting was applied.
Gating strategy	All samples including negative and positive controls were first gated by FSC/SSC, followed by the gating on FSC-W/FSC-A to exclude all the doublets. Finally, the subpopulations were analyzed by Annexin V- APC/7-AAD or gamma-H2AX-Alexa488/ RNH-mCherry, depending on experimental designs.

☐ Tick this box to confirm that a figure exemplifying the gating strategy is provided in the Supplementary Information.
